# Scalable Biomimetic Coaxial Aligned Nanofiber Cardiac Patch: A Potential Model for “Clinical Trials in a Dish”

**DOI:** 10.3389/fbioe.2020.567842

**Published:** 2020-09-16

**Authors:** Naresh Kumar, Divya Sridharan, Arunkumar Palaniappan, Julie A. Dougherty, Andras Czirok, Dona Greta Isai, Muhamad Mergaye, Mark G. Angelos, Heather M. Powell, Mahmood Khan

**Affiliations:** ^1^Department of Emergency Medicine, The Ohio State University Wexner Medical Center, Columbus, OH, United States; ^2^Dorothy M. Davis Heart & Lung Research Institute, The Ohio State University Wexner Medical Center, Columbus, OH, United States; ^3^Centre for Biomaterials, Cellular and Molecular Theranostics, Vellore Institute of Technology, Vellore, India; ^4^Department of Anatomy and Cell Biology, University of Kansas Medical Center, Kansas City, KS, United States; ^5^Department of Biomedical Engineering, The Ohio State University, Columbus, OH, United States; ^6^Department of Materials Science and Engineering, The Ohio State University, Columbus, OH, United States; ^7^Research Department, Shriners Hospitals for Children, Cincinnati, OH, United States; ^8^Department of Physiology and Cell Biology, The Ohio State University Wexner Medical Center, Columbus, OH, United States

**Keywords:** nanofibers, cardiac patch, myocardial infarction, cardiovascular disease, multielectrode array (MEA), 3D model, induced pluripotent stem cell-derived cardiomyocytes

## Abstract

Recent advances in cardiac tissue engineering have shown that human induced-pluripotent stem cell-derived cardiomyocytes (hiPSC-CMs) cultured in a three-dimensional (3D) micro-environment exhibit superior physiological characteristics compared with their two-dimensional (2D) counterparts. These 3D cultured hiPSC-CMs have been used for drug testing as well as cardiac repair applications. However, the fabrication of a cardiac scaffold with optimal biomechanical properties and high biocompatibility remains a challenge. In our study, we fabricated an aligned polycaprolactone (PCL)-Gelatin coaxial nanofiber patch using electrospinning. The structural, chemical, and mechanical properties of the patch were assessed by scanning electron microscopy (SEM), immunocytochemistry (ICC), Fourier-transform infrared spectroscopy (FTIR)-spectroscopy, and tensile testing. hiPSC-CMs were cultured on the aligned coaxial patch for 2 weeks and their viability [lactate dehydrogenase (LDH assay)], morphology (SEM, ICC), and functionality [calcium cycling, multielectrode array (MEA)] were assessed. Furthermore, particle image velocimetry (PIV) and MEA were used to evaluate the cardiotoxicity and physiological functionality of the cells in response to cardiac drugs. Nanofibers patches were comprised of highly aligned core-shell fibers with an average diameter of 578 ± 184 nm. Acellular coaxial patches were significantly stiffer than gelatin alone with an ultimate tensile strength of 0.780 ± 0.098 MPa, but exhibited gelatin-like biocompatibility. Furthermore, hiPSC-CMs cultured on the surface of these aligned coaxial patches (3D cultures) were elongated and rod-shaped with well-organized sarcomeres, as observed by the expression of cardiac troponin-T and α-sarcomeric actinin. Additionally, hiPSC-CMs cultured on these coaxial patches formed a functional syncytium evidenced by the expression of connexin-43 (Cx-43) and synchronous calcium transients. Moreover, MEA analysis showed that the hiPSC-CMs cultured on aligned patches showed an improved response to cardiac drugs like Isoproterenol (ISO), Verapamil (VER), and E4031, compared to the corresponding 2D cultures. Overall, our results demonstrated that an aligned, coaxial 3D cardiac patch can be used for culturing of hiPSC-CMs. These biomimetic cardiac patches could further be used as a potential 3D *in vitro* model for “clinical trials in a dish” and for *in vivo* cardiac repair applications for treating myocardial infarction.

## Introduction

Cardiovascular diseases (CVDs) are the number one cause of morbidity in North America. However, the development of therapeutics for CVDs has been limited by the paucity of efficient model systems for drug screening and toxicology studies (Schroer et al., 2019). Most pharmacological studies make use of primary cell lines or model organisms like rodents, rabbits, pigs, and non-human primates for assessing the effect of putative drug molecules (Savoji et al., 2019). Small animal models like rodents, which have been extensively used for pre-clinical cardiovascular drug testing, are not ideal models, since their cardiomyocytes differ significantly from humans in their structure and function (Milani-Nejad and Janssen, 2014). On the other hand, large animal models (pigs and non-human primates) are good systems as their cardiovascular system and the associated hemodynamics are similar to humans. However, the costs associated with their housing, maintenance, and ethical concerns make them less favorable for pre-clinical drug testing applications (Milani-Nejad and Janssen, 2014). While primary cultures of adult cardiomyocytes are a good, cost-effective system to study drug effects *in vitro*, their use is restrained by their limited availability and lack of efficient culture protocols (Ribeiro et al., 2019).

In this context, human induced-pluripotent stem cell-derived cardiomyocytes (hiPSC-CMs) have become increasingly popular for use as an *in vitro* model system (Karakikes et al., 2015). These cells have shown great potential in developing strategies for cardiac repair (Park and Yoon, 2018). Additionally, the advances in techniques for hiPSC generation, ease of scalability of hiPSC-CMs, and development of next-generation genetic manipulation techniques make hiPSC-CMs an attractive model for the development of patient-specific personalized precision medicine (Park and Yoon, 2018; Gintant et al., 2019; Ribeiro et al., 2019). Hence, hiPSC-CMs have become increasingly popular as an *in vitro* model for cardioprotective and cardiotoxic drug screening (Lynch et al., 2019; Ribeiro et al., 2019). However, in most of these studies, hiPSC-CMs used were cultured in two-dimensional (2D) culture dishes (Antoni et al., 2015; Savoji et al., 2019). The 2D culture system has multiple drawbacks: (a) immature phenotype of hiPSC-CMs, (b) heterogeneity of the cells in culture, (c) lack of alignment of hiPSC-CMs, and (d) inconsistent response to drug treatment (Karakikes et al., 2015; da Rocha et al., 2017). However, recent studies have shown improved function and maturation of hiPSC-CMs in 2D cultures with increased culture time (Kumar et al., 2019) or electrical and mechanical stimulation (Machiraju and Greenway, 2019).

Overall, three-dimensional (3D) culture systems have been shown to improve the maturation as well as functionality of hiPSC-CMs (Savoji et al., 2019). Cells cultured in a 3D environment are shown to have better physiological characteristics and more closely resemble native tissue than the same cells grown in classical 2D culture flasks (Savoji et al., 2019). Hence, 3D cultures provide a better and more relevant model for cardiotoxicity and drug screening studies (Zuppinger, 2016, 2019; Archer et al., 2018; Savoji et al., 2019). The 3D models currently explored as CVD models include: (a) hydrogel-based engineered heart tissue (Weinberger et al., 2017), (b) self-assembling spheroids formed *via* hanging drop method (Figtree et al., 2017), (c) cardiac cell-sheets (Shimizu et al., 2003), and (d) bioengineered scaffolds (Khan et al., 2015, 2018; Kc et al., 2019). Of these, the scaffold-based models have been extensively studied for the development of engineered heart tissues (Gao et al., 2018; Dattola et al., 2019; Jabbour et al., 2019).

Three-dimensional scaffolds have been fabricated using different bioengineering techniques, like microfluidics (Wang et al., 2010), 3D bioprinting (Zhu et al., 2016), gas foaming (Costantini and Barbetta, 2018), and electrospinning (Jun et al., 2018). Of these, electrospinning provides for reduced batch-to-batch variation, better uniformity within scaffolds, nano-dimensional architecture similar to cardiac tissue, and controlled alignment of nanofibers (Kai et al., 2011; Khan et al., 2015; Kitsara et al., 2017). Further, nanofiber-based scaffolds have been fabricated using a wide variety of natural and synthetic biocompatible materials. It has been reported that scaffolds made using natural polymers like gelatin and collagen (Aldana and Abraham, 2017) exhibit efficient cell adhesion but poor mechanical properties. Alternatively, the scaffolds made using synthetic polymers like poly(lactic-co-glycolic) acid (PLGA; Khan et al., 2015), polylactic acid (PLA; Wang et al., 2017), and polycaprolactone (PCL; Chen et al., 2015) have poor biomimetic and cell adhesion properties but improved mechanical support (Bhattarai et al., 2018). In our study, we fabricated an aligned coaxial nanofibrous scaffold, with nanofibers having a PCL core with a gelatin shell. The PCL imparts mechanical strength while gelatin provides the required biomimetic properties, thereby improving cell attachment. The hiPSC-CMs were seeded and cultured on the surface of a 3D aligned coaxial nanofibrous scaffold to obtain a functional 3D ‘cardiac patch,’ which was then used for drug screening and toxicity studies.

## Materials and Methods

### Fabrication of PCL-Gelatin Aligned Coaxial Nanofibrous Patch

**FIGURE 1:**
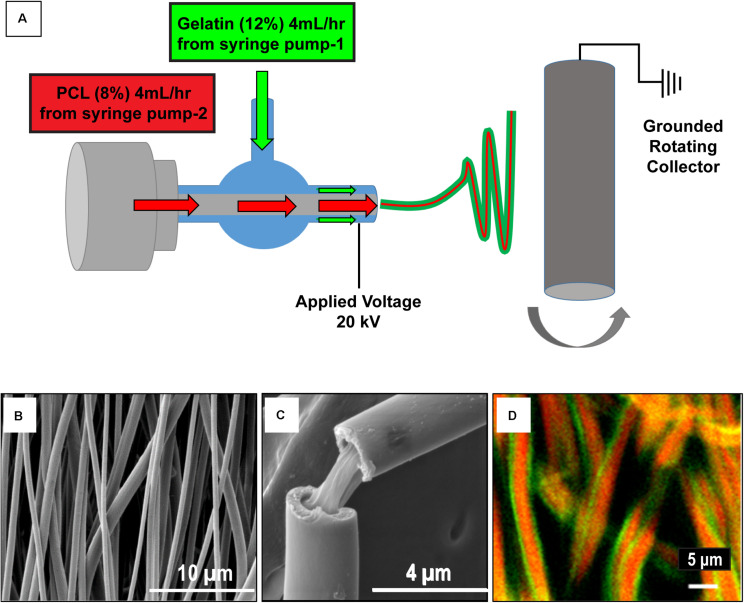
Schematic of the aligned coaxial nanofibrous cardiac patch fabrication process. **(A)** Polycaprolactone (PCL; 8% w/v) and Gelatin (12% w/v) were employed for the fabrication of aligned coaxial (CoA) nanofibrous patches by an electrospinning method. **(B)** SEM image of the aligned CoA nanofibrous patch. **(C)** SEM image of a single CoA nanofiber showing a core-shell structure. **(D)** Confocal image of aligned CoA nanofibers showing the presence of PCL (red) in the core and gelatin (green) in the shell.

Gelatin (12% w/v) [gelatin from bovine skin, Sigma-Aldrich, St. Louis, MO] and PCL (8% w/v) [Sigma-Aldrich, St. Louis, MO; Mn = 42,500] solutions were prepared in 1,1,1,3,3,3-hexafluoro-2-propanol. The gelatin and PCL solutions were fed to the outer (at a flow rate of 4 ml/h) and inner tube (at a flow rate of 1 ml/h), respectively, of the coaxial spinneret as shown in Figure 1A. The distance between the spinneret tip and the grounded rotating collector was maintained at 20 cm and a 20 kV voltage was applied at the spinneret tip. The aligned coaxial nanofibers collected were dried inside a chemical fume hood overnight, to remove remnant solvent.

The morphology of the nanofibrous patch was assessed by scanning electron microscopy (SEM). To confirm the coaxial morphology of the nanofibrous patch, gelatin and PCL solutions were mixed with 1% w/v fluorescein (Sigma-Aldrich, St. Louis, MO) and rhodamine (Sigma-Aldrich, St. Louis, MO), respectively, and nanofibrous patches were fabricated as mentioned above and imaged using a confocal microscope (Olympus FV3000 Confocal microscope).

Patches were first cross-linked, sterilized, and hydrated using the protocol described previously (Drexler and Powell, 2011). Briefly, patches were cut into the desired diameter using biopsy punches (8 mm) and treated with 7 mM 1-ethyl-3-(3Dimethylamino propyl)carbodiimide hydrochloride (EDC) solution in ethanol for 24 h, followed by incubation in 70% ethanol for hydration and sterilization. The patches were then washed in phospahate-buffered saline (PBS), two times for 24 h each. Patches were then used for confocal imaging, mechanical testing, and cell seeding.

### Fourier-Transform Infrared Spectroscopy (FTIR) Studies

Surface chemical analysis of the gelatin-only, PCL-only, and coaxial patches was performed using attenuated total reflection Fourier transform infrared spectroscopy (ATR-FTIR) between the range of 400–4000 cm^–1^ (Thermo Nicolet Nexus 670 FTIR spectrometer, MN). Approximately 25–40 scans were performed on the three types of patches.

### Mechanical Testing of Nanofiber Scaffolds

The mechanical properties of acellular aligned PCL, gelatin, and PCL-gelatin coaxial nanofibrous patches were tested using a TestResources 100R (Shakopee, MN) as per the American Society for Testing and Materials (ASTM) D638 Type V standard, using the protocol described previously (Johnson et al., 2007; Blackstone et al., 2018). For aligned gelatin and coaxial patches, tests were performed after crosslinking in EDC solution and further hydration in PBS (described above). For aligned PCL patches, incubation was carried out in ethanol (to mimic the crosslinking process) before hydration in PBS. The thickness of each sample was measured using digital calipers. Samples were strained (along the axis of alignment) at a grip speed of 2 mm/s until failure. Samples were made using a dog-bone shaped punch, 3 mm gauge width, and 10 mm gauge length. The thickness of each sample was quantified using digital calipers. Stress-strain curves were generated for each sample and the ultimate tensile strength and Young’s modulus were determined and reported as mean ± SD. Ultimate tensile strength was determined at the point of greatest stress, before failure. Young’s modulus was calculated from the stress-strain curve using linear regression analysis for the first linear region of the curve (where *R*^2^ ∼0.98) past the toe-in region, if present.

### Degradation Studies

The scaffold was cut into small 8 mm diameter circular patches and incubated in medium at 37°C for 2 weeks. After 1 and 2 weeks, the patches were rinsed in water, air-dried and weighed to determine their weight loss by degradation. The weights of four independent samples were measured for each time point. Additionally, the patches were imaged using SEM to assess any morphological changes in the fibers during the 2-week culture duration.

### Culturing and Maintenance of hiPSC-CMs on Aligned Coaxial Nanofibrous Patches

The hiPSC-CMs were obtained from Fujifilm Cellular Dynamics International (CDI, Madison, WI, United States, Cat# R 1007). The cells were plated in sterile six-well plates according to the manufacturer’s protocol and after 48 h, cells were cultured in CDI hiPSC-CMs maintenance medium in a humidified atmosphere at 37°C at 5% CO_2_, as previously described (Citro et al., 2014; Khan et al., 2015).

Once the hiPSC-CMs showed contractions, the cells were seeded onto aligned coaxial nanofibrous patches. For this, sterile cross-linked 8 mm aligned coaxial patches were transferred onto N-terface (Winfield Labs, Richardson, TX, United States) and coated with 30 μl of fibronectin (50 μg/ml) for 1 h in a humidified atmosphere at 37°C. The patches on N-terface were then transferred onto sterile sponges soaked in the hiPSC-CMs’ culture medium placed in a 100 mm culture dish. The hiPSC-CMs were harvested using 0.25% trypsin-EDTA and seeded onto the coaxial patches at a final density of 1 × 10^6^ cells/cm^2^ (50 μl of cell suspension/patch) and incubated at 5% CO_2_ at 37°C for 1 h. After 4–6 h, the aligned coaxial cardiac patches (aligned coaxial nanofibrous patches seeded with hiPSC-CMs) were transferred to six-well plates (one patch/well) containing 2 ml CDI hiPSC-CMs maintenance medium. The medium was replaced every alternate day for 2 weeks.

### Scanning Electron Microscopy (SEM)

Scanning electron microscopy was used to observe the morphology of the aligned PCL, gelatin, and PCL-gelatin coaxial nanofibrous patches. SEM was also performed on aligned coaxial cardiac patches to determine the distribution and alignment of the seeded hiPSC-CMs. For these studies, the samples were prepared as described previously (Huang et al., 2013; McBane et al., 2013; Khan et al., 2015). In brief, the cardiac patches were fixed in 4% paraformaldehyde (PFA, MilliporeSigma, Milwaukee, WI, United States) for 1 h. Patches were then washed with de-ionized water and further dehydrated by gradually increasing ethanol concentration (50, 70, 80, 95, and 100%). The patches were then dried chemically using an increasing gradient of hexamethyldisilazane. Finally, patches were sputter-coated with gold-palladium (Pelco Model 3) and imaged on FEI NOVA nanoSEM.

### Cellular Viability Staining of hiPSC-CMs

LIVE/DEAD^TM^ Cell Imaging Kit (Cat # R37601) was procured from Invitrogen (Life Technologies Corporation, Carlsbad, CA, United States) and used to stain the live and dead cells in culture per the manufacturer’s instructions. After staining, the hiPSC-CMs were washed with maintenance medium three times and imaging was performed on an EVOS^TM^ FL Auto 2 Fluorescent microscope (Thermo Fisher Scientific, Waltham, MA, United States).

### Lactate Dehydrogenase (LDH) Assay

To measure the cytotoxicity of the aligned coaxial patch on hiPSC-CMs, LDH assay was performed using the *in vitro* toxicology assay kit, lactic dehydrogenase based (Cat# TOX7-1KT, MilliporeSigma, Milwaukee, WI, United States). The culture medium was collected from hiPSC-CMs cultured in tissue culture plates or on aligned coaxial patches after 48 h of culture and LDH release assay was performed according to the manufacturer’s protocols. Background and primary absorbance of the plate were measured on a spectrophotometer (2030 Multilabel Reader, Victor^TM^ ×3, PerkinElmer, Inc., Waltham, MA, United States) at 690 and 490 nm, respectively. The assay was performed in quadruplicate (*n* = 4) and data obtained was analyzed on WorkOut 2.5 (build 0428, PerkinElmer, Inc., Waltham, MA, United States), by subtracting background absorbance from primary absorbance.

### Immunostaining for Cardiac Markers

As described previously (Khan et al., 2015), immunofluorescence staining was performed to analyze the expression of cardiac markers in aligned coaxial cardiac patches. Briefly, aligned coaxial cardiac patches, after 2 weeks in culture, were washed twice with PBS and fixed with 4% PFA for 10 min at room temperature. The patches were then washed twice with PBS and incubated in blocking buffer (PBS, 5% normal goat serum, and 0.3% Triton X) for 1 h to block non-specific antibody binding. Following this, the patches were incubated with anti-α-sarcomeric actinin (A7811, MilliporeSigma, Milwaukee, WI, United States), anti-GATA4 (PA1-102, Thermo Fisher Scientific, Waltham, MA, United States), anti-Troponin-T (HPA017888, MilliporeSigma, Milwaukee, WI, United States), and anti-Connexin-43 (MAB 3067, MilliporeSigma, Milwaukee, WI, United States) antibodies, overnight at 4°C and after which the patches were washed thrice in PBS, 5 min. each. Cells were then incubated with the corresponding secondary antibodies conjugated either with Texas Red or FITC against rabbit (1:5000, 8889S, Cell Signaling Technology, Danvers, MA, United States) or mouse (1:5000, 4408S, Cell Signaling Technology, Danvers, MA, United States) for 1 h at room temperature in the dark and washed thrice in PBS. Nuclei were counterstained with NucBlue (R37605, Invitrogen, Carlsbad, CA, United States). Finally, the patches were washed thrice with PBS, transferred onto slides and mounted with ProLong^TM^ Glass Antifade mounting medium (Cat# P36984, Invitrogen, Carlsbad, CA, United States). Imaging was performed on a confocal microscope (Olympus FV 1000 spectral, Olympus Corporation, Center Valley, PA, United States) and images were processed using the Olympus FLUOVIEW Ver. 4.2a Viewer.

### Assessment of Calcium Cycling in hiPSC-CMs

Calcium transients were imaged in hiPSC-CMs cultured on fibronectin-coated glass coverslips and aligned coaxial cardiac patches using the calcium-binding dye Fluo-3, AM (F1242, Invitrogen, Carlsbad, CA, United States). For staining, the hiPSC-CMs cultured on coverslips or patches were washed three times with Dulbecco’s Modified Eagle’s Medium (DMEM) and incubated in 5 μM Fluo 3-AM in DMEM for 1 h in dark at 37°C, 5% CO_2_ in a humidified atmosphere. The cells were then washed three times in DMEM and incubated for an additional 30 min in serum-containing medium at 37°C, 5% CO_2_ in a humidified atmosphere. The cell culture plate was then placed on the microscope (Leica Microsystems, Wetzlar, Germany) stage to record a movie using Leica Application Suite X 3.0.6.17580 software.

### Particle Image Velocimetry (PIV)

#### Cross-Correlation (“PIV”) Analysis of Images

Cell contractility kinetics were assessed using the optical flow/PIV method described previously (Zamir et al., 2005; Aleksandrova et al., 2012). Briefly, a motion pattern (velocity field) captured on a pair of images was calculated by dividing the first image into overlapping tiles, each 64 pixels wide. The second image was then scanned pixel-by-pixel, by shifting an equally sized (64 pixels × 64 pixels) window. The most similar (by Euclidean distance) tile on the second image was then assumed to be the location where the pattern in the first image moved. The resulting displacement vectors characterizing each image tile were then interpolated and denoised by a thin-plate spline fit, yielding our coarse displacement field. The coarse estimate was used to construct a second, higher resolution displacement field. In this second step, the cross-correlation search for pattern similarity was repeated with tiles that were only 32 pixels wide but in a much smaller search area allowing only for four-pixel displacements around the location predicted by the coarse displacement field.

#### Beat Patterns

To determine the beat patterns, a suitable reference image taken at time *t*^∗^, which is a frame between two contraction cycles, where movement is minimal: *V*(*t*) ≥ *V*(*t*^∗^), in a motion-free state, was first identified. This reference image was then compared to all other images of the recordings with cross-correlation (“PIV”) analysis. The result is a series of displacement vector fields *d*(*t*,*x*), which estimate for each time point *t* and location *x* the total movement (magnitude and directionality) relative to a resting state. For each time point *t* the beat pattern *D*(*t*) is the spatial average of the magnitude of *d*(*t*,*x*) as *D*(*t*) ≤ |*d*(*t*,*x*)|>*_x_*, where < … > *_x_* denotes spatial averaging over all possible locations *x*.

#### Frequency Analysis

Fourier spectra were calculated from *D*(*t*) beat patterns using the discrete Fourier transform algorithm as described previously (Rajasingh et al., 2018). Power densities were calculated as the magnitudes of the squared Fourier spectra, and indicate periodicity within the signal in the form of peaks at the corresponding frequencies. When the analyzed signal is not a pure sine wave, harmonics are expected to appear at integer multiples of the fundamental frequency *f* (*2f*, *3f*, etc.). The magnitude of a peak in the power spectrum indicates the amplitude of the signal oscillating with the corresponding frequency.

#### Convergence Analysis

Cell layers often move passively without actively contracting. The optical flow-based method does not distinguish between active and passive (elastic response of the adjacent cell layer) contractility. To identify contractile centers, we estimated the convergence maps of the displacement field as its negative divergence from optical flow data *d*(*t,x*) as previously described (Czirok et al., 2017).

### Functional Characterization Using a Multi-Electrode Array (MEA) System

The field potentials of hiPSC-CMs cultured in 2D or on aligned coaxial patches (3D) were measured using an MEA system. For this, hiPSC-CMs were either cultured directly on 24-well MEA plates (M384-tMEA-24W, Axion Biosystems, Atlanta, GA, United States) having 16 PEDOT microelectrodes per well (as described previously; Kumar et al., 2019) or on 8 mm aligned coaxial patches; both were cultured for 2 weeks. The aligned coaxial cardiac patches were transferred into a sterile six-well MEA plate (M384-tMEA-6W, Axion Biosystems, Atlanta, GA, United States) having 64 PEDOT microelectrodes per well. The plate was equilibrated in the MEA system (Maestro Edge, Axion Biosystems, Atlanta, GA, United States) for 30 min in 5% CO_2_ with a humidified atmosphere at 37°C. For the patches, the excess culture medium was removed to facilitate better contact with the electrodes. The baseline was recorded for each well for 5 min. After which the hiPSC-CMs in 2D, as well as the cardiac patches, were treated with different cardiac drugs: (a) Isoproterenol (ISO, 10 and 100 nM), (b) Verapamil (VER, 0.1 and 0.3 μM), and (c) E4031 hydrochloride (E4031, 50 and 100 nM). The stock solutions of all drugs were prepared in dimethylsulphoxide (DMSO). The plates were equilibrated for 5 min after the addition of drugs and the field potentials were recorded for 5 min for each drug treatment. AxIS Navigator version^TM^ 1.4.1.9 was used for data recording while CiPA^TM^ analysis tool version 2.1.10 (Axion Biosystems, Atlanta, GA, United States) was used for data analysis. The beat period, field potential duration (FPD), spike amplitude, and the incidences of arrhythmias were calculated. Further, the Fredericia’s correction was applied to the FPD, to interpret the effect of drugs on the QT interval. Data are expressed as mean ± SD (*n* = 3).

### Statistical Analysis

Data acquired is expressed as mean ± SD. Statistical significance was determined using one-way ANOVA. All pairwise multiple comparison procedures were performed by the Holm–Sidak method. *p*-value < 0.05 was considered statistically significant.

## Results

### Fabrication of Aligned Nanofibrous Coaxial, PCL, and Gelatin Patches

**FIGURE 2:**
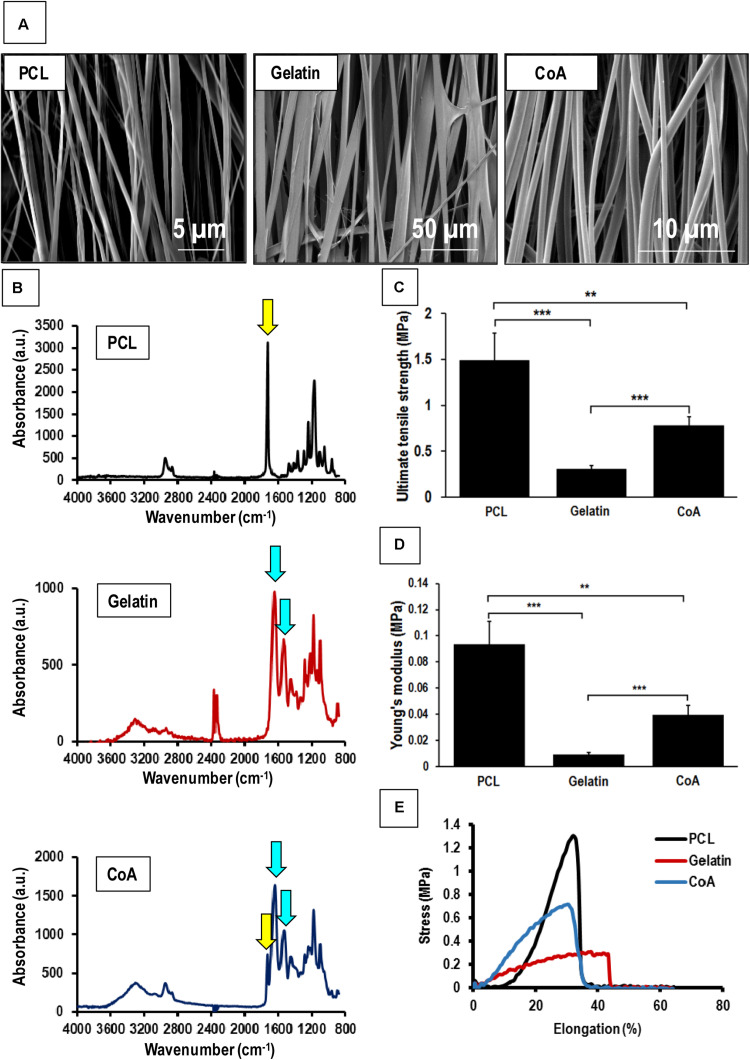
Mechanical testing of aligned nanofibrous patches and comparison with aligned coaxial patch. **(A)** SEM images of aligned PCL, aligned gelatin, and aligned coaxial (CoA) PCL/Gel nanofibrous patches. **(B)** FTIR spectra of aligned PCL, aligned gelatin, and aligned CoA PCL/Gel nanofibrous patches. **(C)** Tensile strength, yellow and blue arrows indicate functional groups corresponding to PCL and gelatin, respectively, **(D)** Young’s modulus, and **(E)** Stress vs. elongation% for aligned PCL, aligned gelatin, and aligned CoA PCL/Gel nanofibrous patches. n = 4; **p < 0.01; ***p < 0.001.

Aligned nanofibrous PCL-gelatin coaxial patches were successfully fabricated via electrospinning (Figure 1A). Coaxial nanofibers were highly aligned with a mean diameter of 578 ± 184 nm (Figure 1B). The overall mean thickness of the aligned coaxial nanofibrous patches was 115 ± 11 μm. SEM imaging of a single nanofiber showed a core-shell structure indicating successful coaxial morphology (Figure 1C). Further, confocal microscope images of the coaxial patches after mixing of rhodamine and fluorescein with PCL and gelatin, respectively, validated the coaxial morphology. These images clearly showed the presence of PCL (red) in the core and gelatin (green) in the shell (Figure 1D). Following hydration, confocal image analysis showed that the nanofibers had a diameter of 2.21 ± 0.50 μm. Additionally, a comparison of the SEM images of pure PCL and pure gelatin nanofibrous patches versus coaxial patches clearly showed that the coaxial nanofibers were more uniform and cylindrical in morphology (Figure 2A). AFTIR analysis of the PCL and gelatin patches showed peaks corresponding to C = O ester of PCL at 1722 cm^–1^ (green) and peaks corresponding to the amide groups of gelatin at 1544 and 1657 cm^–1^ (brown; Figure 2B). Coaxial patches exhibited all three peaks (Figure 2B), further reiterating the presence of both polymers in the nanofibers.

### Mechanical Testing of Aligned Coaxial Patches

The mechanical strength of PCL, gelatin, and the PCL-gelatin coaxial patches was determined by tensile testing. The stress-strain curves obtained showed that the aligned PCL-only patch had the highest values for both tensile strength (1.490 ± 0.290 MPa) as well as Young’s modulus (0.093 ± 0.017 MPa; Figures 2C,D). This was followed by the aligned coaxial patch, which had a tensile strength and Young’s modulus of 0.780 ± 0.098 and 0.039 ± 0.007 MPa, respectively (Figures 2C,D). The aligned gelatin patches had the least mechanical strength with tensile strength and Young’s modulus of 0.308 ± 0.032 and 0.009 ± 0.001 MPa (Figures 2C,D), respectively. However, in terms of percent elongation, gelatin showed the highest elongation, while no significant difference was observed between the PCL and coaxial patches (Figure 2E). Therefore, the aligned coaxial patches had strength and stiffness intermediate to PCL and gelatin, a percent elongation at failure similar to PCL alone, and their elongation at failure matched the PCL patches.

### Degradation of Aligned Coaxial Nanofiber Scaffolds

**FIGURE 3:**
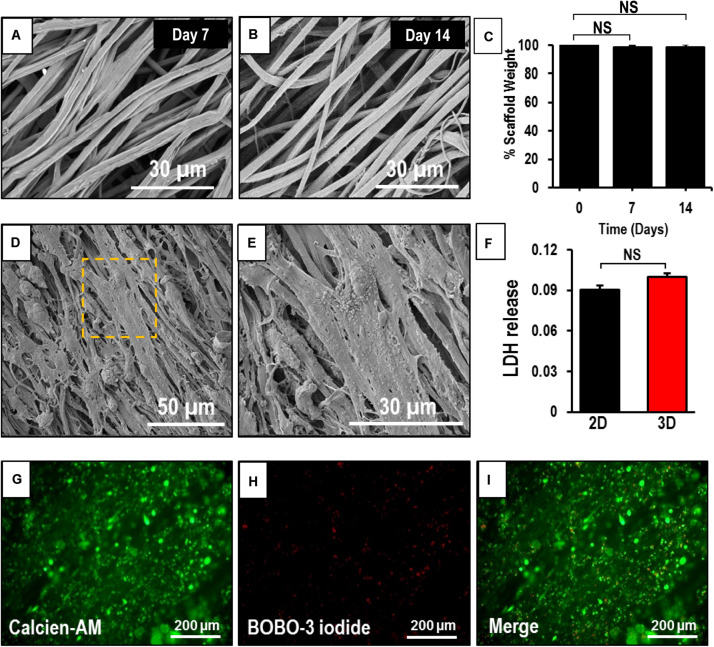
Assessment of degradability and biocompatibility of human induced-pluripotent stem cell-derived cardiomyocytes (hiPSC-CMs) seeded on an aligned coaxial patch. **(A,B)** SEM images of the aligned coaxial patches after 1 and 2 weeks in culture. **(C)** Weight of the coaxial scaffolds after 1 and 2 weeks in culture. NS, not significant. **(D,E)** SEM images of hiPSC-CMs cultured on aligned coaxial (CoA) PCL/Gel nanofibrous patches. **(F)** Fold change in LDH released by hiPSC-CMs cultured in tissue culture plates (2D) and CoA patches (3D) as compared control. Data expressed as mean ± SD, *n* = 3. NS, not significant. **(G–I)** Live-dead cell staining of hiPSC-CMs cultured on coaxial aligned scaffolds showing live cells stained with Calcein-AM (green) and dead cells stained with BOBO-3 iodide (red) after 2 weeks in culture.

The degradation of the coaxial scaffolds at 1 and 2 weeks was determined by measuring their weight loss and studying their morphology. SEM images of the scaffolds after 7 and 14 days (Figures 3A,B) in culture did not show any striking differences when compared to the crosslinked patches at the beginning of the experiment (Figure 1B). Furthermore, no significant difference was observed in the weight of the scaffold after 7 and 14 days in the culture (Figure 3C). Taken together, this data suggests that the scaffolds did not undergo rapid degradation under the culture conditions used in our study.

### Morphology and Viability of hiPSC-CMs on Aligned Coaxial Nanofibrous Patches

The hiPSC-CMs cultured on aligned coaxial patches and their morphology was assessed at 2 weeks by SEM. The SEM images showed a uniform distribution and attachment of the hiPSC-CMs on the coaxial patches (Figure 3D). The hiPSC-CMs also showed a parallel alignment with the nanofibers (Figure 3E). The viability of the hiPSC-CMs cultured on the coaxial patches was assessed by staining the cardiomyocytes with Calcein-AM (for live cells), BOBO-3 iodide (for dead cells), and LDH assay (Figure 3F). Fluorescence images showed that a majority of cardiomyocytes stained positive for calcein-AM, indicating that they are viable and metabolically active (Figures 3G,I). Additionally, some cardiomyocytes stained positive for BOBO-3 iodide (Figures 3H,I), indicating that the patch was biocompatible. Furthermore, no significant differences were observed in LDH released from hiPSC-CMs cultured on the aligned coaxial nanofibrous patches (3D) versus on a flat-plate (2D) (Figure 3F). Taken together, these results showed that aligned coaxial PCL/Gel patches are biocompatible and support the 3D culture of hiPSC-CMs.

### Confocal Imaging to Assess the Expression of Cardiac Markers in hiPSC-CMs Cultured on an Aligned Coaxial Patch

**FIGURE 4:**
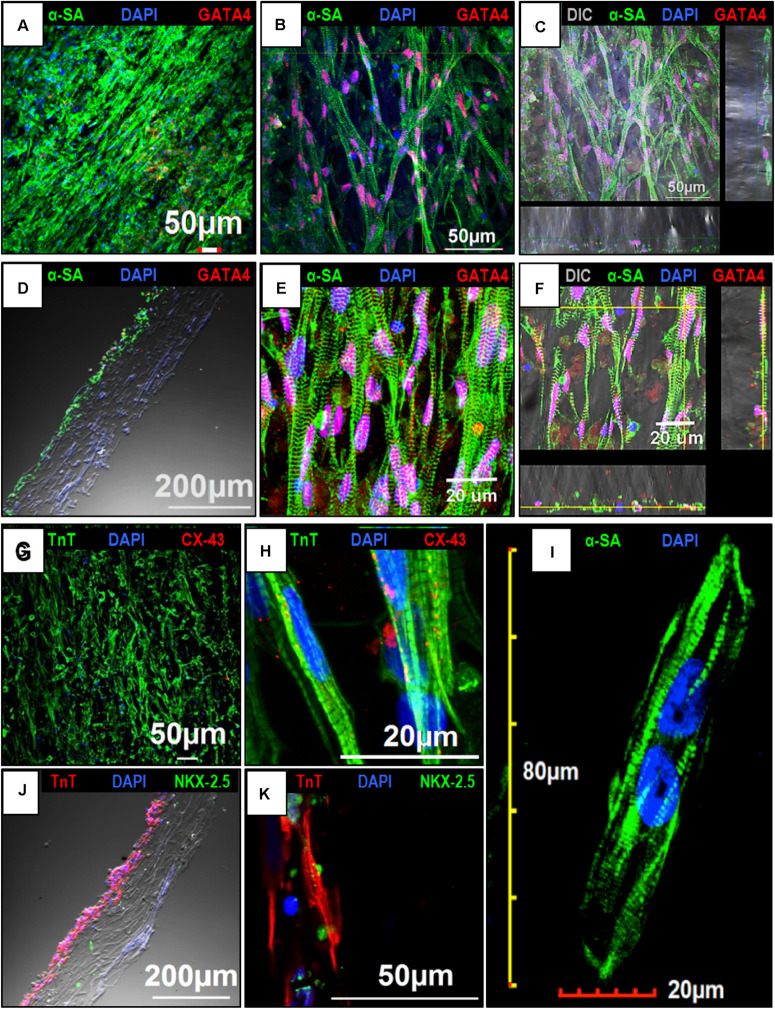
Confocal imaging to assess the expression of cardiac markers in human induced-pluripotent stem cell-derived cardiomyocytes (hiPSC-CMs) cultured on the aligned coaxial nanofiber patch. **(A–F)** Confocal images showing the expression of α-SA and GATA4 in hiPSC-CMs cultured on aligned coaxial patches. **(G,H)** Confocal images showing the expression of TnT and Cx-43 in hiPSC-CMs cultured on aligned coaxial patches. **(J,K)** Confocal images showing the expression of TnT and Nkx2.5 in hiPSC-CMs cultured on aligned coaxial patches. **(C,F)** Z-stack images showing the distribution of the hiPSC-CMs through the depth of the coaxial patches. **(D,J)** Cross-section images of the coaxial patches showing the distribution of hiPSC-CMs in the patch. **(I)** Confocal image of a single hiPSC-CM cultured on an aligned coaxial patch.

The expression of cardiac makers in hiPSC-CMs was assessed by confocal imaging to understand the intracellular sarcomere arrangement in the cells following culture on the aligned coaxial patches. At 2 weeks, the hiPSC-CMs cultured on aligned coaxial patches stained positive for the cardiac lineage markers; GATA 4, Nkx2.5, α-sarcomeric actinin (α-SA), cardiac Troponin T (TnT), and connexin-43 (Cx-43) (Figure 4). Expression of the cardiac transcription factors GATA4 (Figures 4A–F) and Nkx2.5 (Figures 4J,K) was detected in the nucleus of the hiPSC-CMs. Additionally, these hiPSC-CMs showed parallelly arranged sarcomeres, as observed by α-SA (Figures 4A–F,I) and TnT staining (Figures 4G,H,J,K). Also, the hiPSC-CMs showed the expression of Cx-43, indicating good intercellular contact between neighboring cardiomyocytes (Figures 4G,H). The distribution of the hiPSC-CMs was also assessed across the depth of the patch. The cross-sections and Z-stack confocal images of aligned coaxial cardiac patches showed migration of the hiPSC-CMs up to 40–50 microns below the surface of the scaffold (Figures 4C,D,F,J), evidenced by the expression of α-SA, TnT, GATA4, and Nkx2.5. Additionally, increased magnification of a single hiPSC-CM imaged on the patch showed a multi-nucleated rod-shaped morphology with well-organized sarcomeres (Figure 4I). These observations indicated that the aligned coaxial nanofibrous patch can be used as a model for understanding the structural maturation of hiPSC-CMs on 3D scaffolds.

### Assessment of Calcium Transients in hiPSC-CMs Seeded on an Aligned Coaxial Cardiac Patch

**FIGURE 5:**
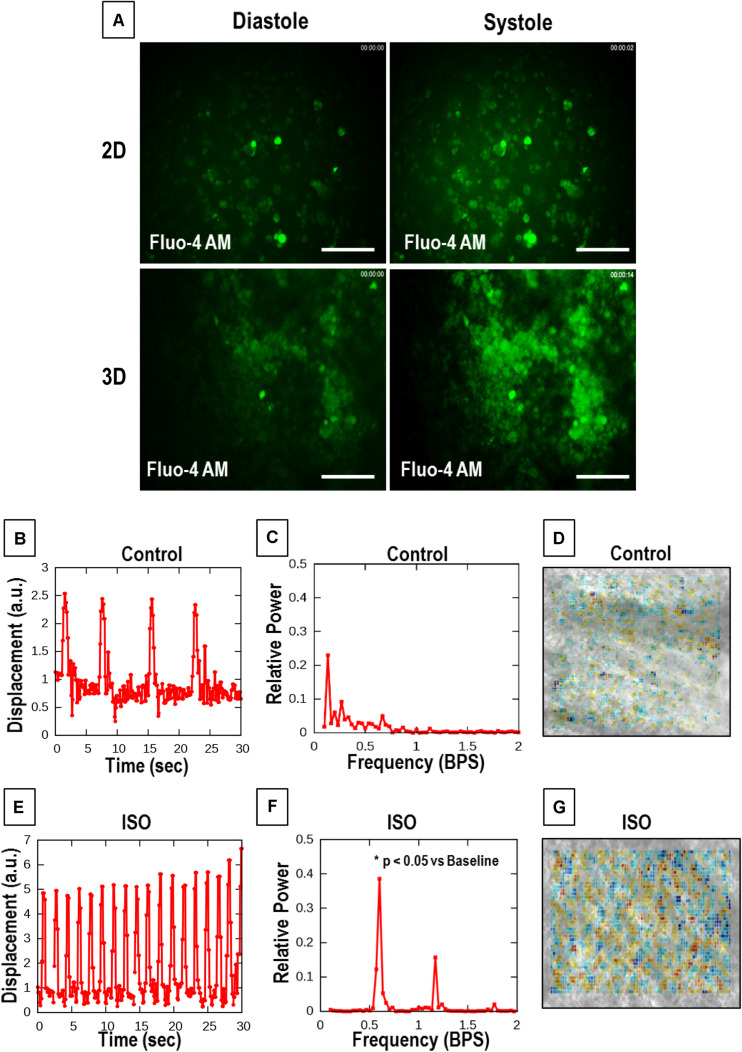
Calcium cycling and optical contractility analysis of the aligned coaxial cardiac patch. **(A)** Representative images of calcium transients in 2D and 3D cultures. **(B–D)** Representative data of untreated control aligned coaxial (CoA) cardiac patches **(B–D)**, and **(E–G)** aligned CoA cardiac patches treated with 100 nM Isoproterenol (ISO). **(B,E)** Spontaneous beat patterns expressed as displacement relative to a resting reference state vs. time. **(C,F)** Fourier power spectra of beat patterns show the dominant beat frequency as a peak and spatially resolved contractility analysis. **(D,G)** Representation of particle image velocimetry (PIV) with warmer colors corresponding to higher divergence, a numerical estimate of contractile strength.

The calcium transients in hiPSC-CMs cultured on tissue culture plates (2D) and aligned coaxial patches (3D) were assessed after 2 weeks in culture. The hiPSC-CMs cultured in 2D and 3D showed synchronous calcium transients (Figure 5A). This data showed that the hiPSC-CMs cultured on aligned coaxial patches formed a functional syncytium as indicated by synchronous calcium waves.

### Assessment of Cell Contractility in hiPSC-CMs Cultured on Aligned Coaxial Patches

Particle image velocimetry analysis was performed to evaluate the contractility of hiPSC-CMs cultured on aligned coaxial patches. For this, the response of the patches, at 2 weeks of culture, to 100 nM ISO, a non-specific β-adrenoreceptor agonist, was analyzed by an optical measure of contractility (Figures 5B–G). For both treated and control cultures, six videomicroscopic recordings were obtained from two parallel cultures. Beat patterns (Figures 5B,E) indicated an ISO-induced increase in spontaneous beat frequency and amplitude. Also, Fourier power spectra of the beat patterns indicated an increase in frequency by shifting the dominant peak toward higher frequencies (right) after ISO treatment (Figures 5C,F). A significant increase (*p* < 0.0004) in the average frequency of spontaneous beating from 0.136 ± 0.0069 to 1.497 ± 0.0394 SEM Hz was observed following ISO treatment (Figures 5C,F). Additionally, ISO treatment also promoted better-defined beating activity that is indicated by the tall narrow peaks. The contractility maps showed a stronger, spatially extended beating activity in ISO-treated patches (Figures 5D,G). These results demonstrate the ability of the aligned coaxial patches to respond to cardiac drugs in a reproducible manner.

### Electrophysiological Assessment of hiPSC-CMs Cultured on Aligned Coaxial Patches

**FIGURE 6:**
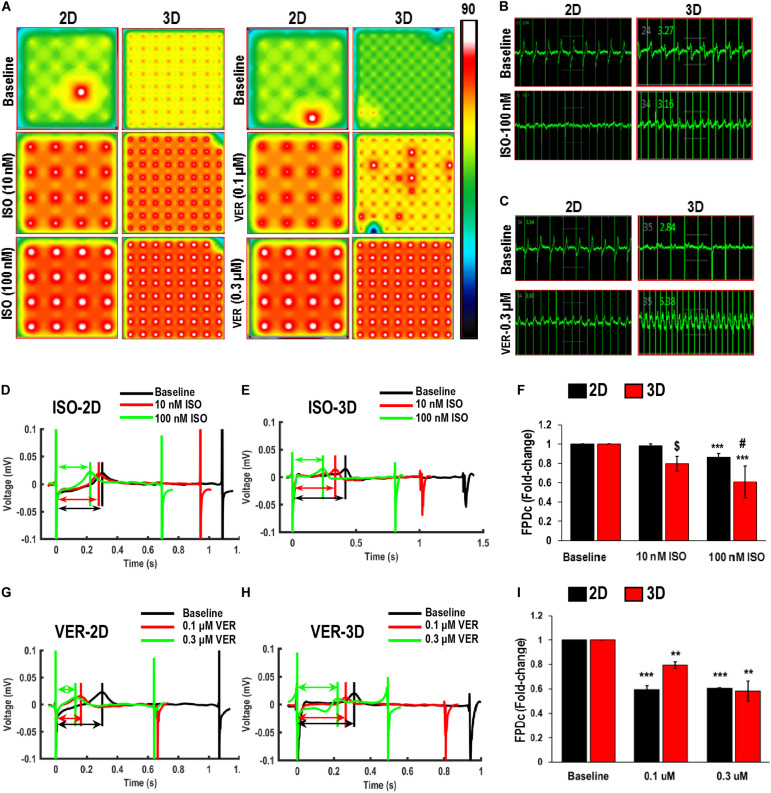
Functional assessment of human induced-pluripotent stem cell-derived cardiomyocytes (hiPSC-CMs) cultured in 2D and 3D cultures in response to Isoproterenol (ISO) and Verapamil (VER). **(A)** Heat maps showing the beat rate in representative wells of 2D and 3D cultures before treatment (baseline) and after treatment with ISO or VER at different concentrations. Representative images showing changes in beat detection before (baseline) and after treatment with **(B)** 100 nM ISO and **(C)** 0.3 μM VER. Representative images showing changes in beat period in **(D,G)** 2D and **(E,H)** 3D cultures after treatment with **(D,E)** ISO and **(G,H)** VER. Quantitative assessment of change in corrected field potential duration (FPDc) after treatment with **(F)** ISO and **(I)** VER, respectively, in 2D and 3D cultures. Dotted arrows in **(D,E,G,H)** show field potential duration (FPD). Scale in A represents the beats per minute. Data shown in **(F,I)** is mean ± SD, *n* = 8, from three independent cultures. **p < 0.01, ***p < 0.001 vs. corresponding baseline; ^[*dollar*]^*p* < 0.001, ^#^*p* < 0.01 vs. corresponding 2D cultures.

**FIGURE 7:**
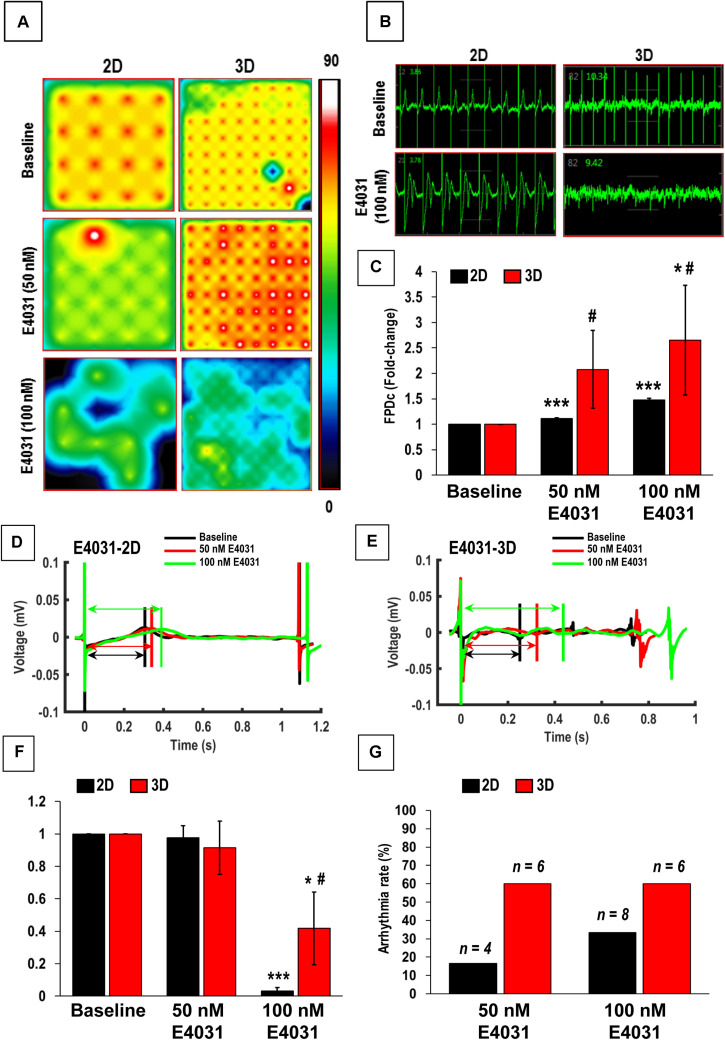
Functional assessment of human induced-pluripotent stem cell-derived cardiomyocytes (hiPSC-CMs) cultured in 2D and 3D cultures after treatment with E4031. **(A)** Heat maps showing the beat rate in representative wells of 2D and 3D cultures before treatment (baseline) and after treatment with E4031. **(B)** Representative images showing changes in beat detection before (baseline) and after treatment with E4031. **(C)** Quantitative assessment of fold change in corrected field potential duration (FPDc) following E4031 treatment in 2D and 3D cultures. Representative images showing changes in beat period in **(D)** 2D and **(E)** 3D cultures after treatment with E4031. Quantitative assessment of change in **(F)** spike amplitude and **(G)** arrhythmicity after E4031 treatment in 2D and 3D cultures. Arrows in **(D,E)** show field potential duration (FPD). Scale in A represents the beats per minute. Data shown in **(C,F,G)** is mean ± SD, *n* = 8, from three independent cultures. *p < 0.05, ***p < 0.001 vs. corresponding baseline; ^#^*p* < 0.01 vs. corresponding 2D cultures.

The hiPSC-CMs cultured in 2D and 3D (aligned coaxial patch) was assessed on the MEA system for field potentials, which result from spontaneous cardiac action potentials propagating across cells on neighboring electrodes. The hiPSC-CMs were treated with different concentrations of ISO, VER, and E4031, and the changes in their field potential was measured. An increase in the beating frequency (beats per minute) was observed in both the 2D as well as 3D cultures following treatment with ISO (Figures 6A,B) and VER (Figures 6A,C), while a decrease in beating frequency was observed following E4031 treatment (Figures 7A,B). These changes were as expected for these drugs’ mechanisms of action.

A significant, dose-dependent decrease in the beat period was observed in hiPSC-CMs in both 2D and 3D cultures after treatment with ISO. After treatment with 10 nM ISO, the beat period in the 2D and 3D cultures decreased from 1.04 ± 0.04 and 1.22 ± 0.16 s to 0.67 ± 0.04 and 0.8 ± 0.15 s, respectively (Figures 6D,E). Similarly, after treatment with 100 nM ISO, the beat period of the hiPSC-CMs in 2D and 3D cultures was 0.66 ± 0.03 and 0.66 ± 0.1 s, respectively (Figures 6D,E), indicating that the dose-dependent response was improved in case of 3D cultures. Furthermore, as expected, ISO treatment resulted in a dose-dependent shortening of the QT interval, as evidenced by the decrease in Friedrica’s corrected field potential duration (FPDc) in both the 2D and 3D cultures (Figures 6D–F). Also, between the 2D and 3D cultures, a significant difference in the FPDc was observed after treatment with 10 and 100 nM ISO, indicating that the 3D cultures responded more robustly to the treatment (Figure 6F). A similar decrease in the beat period and FPDc was observed in the hiPSC-CMs cultured in 2D and 3D cultures after treatment with the L-type Ca channel blocker VER (Figures 6C,G–I). A significant fold decrease in the beat period was observed in both the 2D as well as 3D cultures after treatment with 0.1 and 0.3 μM VER, as compared to their respective baseline controls (Figure 6I). However, a dose-dependent decrease in the FPDc was observed only in the case of the 3D culture indicating that the 3D culture system was a more responsive model for drug testing (Figure 6I).

We also assessed the effect of the hERG-type K^+^ channel blocker E4031, which is an anti-arrhythmic drug. However, this drug is pro-arrhythmic *in vitro* especially at higher doses (Goto et al., 2018). As expected, following treatment with the E4031, a significant increase in the beat period and FPDc was observed in the 2D cultures following treatment with 50 and 100 nM of E4031, while a significant increase was observed in 3D cultures only after treatment with 100 nM E4031 (Figures 7C–E). Furthermore, we also observed higher variability in the FPDc in 3D cultures as compared to the 2D cultures (Figure 7C). This was reiterated by a Comprehensive *in vitro* Pro-arrhythmia Assay (CiPA), which showed that 16.67% (4/24) and 33% (8/24) of the samples in 2D cultures showed beat period irregularity after treatment with 50 and 100 nM E4031, respectively, while patches showed beat period irregularity of 60% following treatment with E4031(6/10). This data indicated the increased occurrence of arrhythmias in these cardiac patches (Figure 7G) making them a more powerful system for arrhythmia detection. Additionally, a significant decrease in the spike amplitude was observed in both the 2D and 3D cultures, following treatment with 100 nM E4031 (Figure 7F). Taken together, these results showed that while hiPSC-CMs cultured in 2D and 3D cultures responded to drug treatments, the 3D cultured hiPSC-CMs had more efficacious responses and would be a better model for *in vitro* drug testing.

## Discussion

The main focus of the present study was to develop a potent and efficient 3D culture system for cardiac drug testing. Our findings demonstrate that aligned PCL-Gel coaxial nanofibrous scaffolds successfully culture hiPSC-CMs in a 3D microenvironment. The comparative assessment of hiPSC-CMs cultured in 2D and 3D culture systems carried out in the present study showed improved functional characteristics and increased responsiveness to cardiac drugs in the latter.

Coaxial PCL-gelatin scaffolds demonstrated excellent biological properties including cell attachment, organization, and expression of cardiac lineage markers along with high mechanical strength and Young’s modulus allowing for the scaffold to be handled easily. Our observations are consistent with previous reports, which have shown coaxial electrospinning is an efficient strategy for surface modification of nanofibrous scaffolds made from synthetic polymers like PCL, PLGA, and polyvinyl alcohol (PVA) (Kim and Cho, 2016). While coaxial electrospinning has been more commonly used for controlled drug/biomolecule release (Zhang et al., 2006; Ji et al., 2010; Sun et al., 2019), it has also been used to coat synthetic polymer-based nanofibers with a natural polymer (like gelatin, alginate, and collagen) to make them more biomimetic (Zhang et al., 2007; Hu et al., 2020). The use of coaxial nanofibers with a PCL inner core and gelatin outer shell has previously been used for wound healing (Blackstone et al., 2014) and vascular (Coimbra et al., 2017) and bone (Alissa Alam et al., 2020) tissue engineering applications. This coaxial structure has been shown to improve the biocompatibility of the scaffolds as well as provide structural support to the cells for *in vivo* applications.

Another striking observation made in our study was the alignment of hiPSC-CMs with the nanofibers in the scaffold. As a result, the hiPSC-CMs cultured on the aligned scaffolds showed an elongated rod-shaped morphology. Additionally, the sarcomeres in these hiPSC-CMs showed parallel organization inside the cell, with some cells being binucleated. These observations indicate the maturation of the cells cultured on the coaxial scaffolds. Our observations are consistent with previous reports demonstrating a similar alignment of hiPCMs when cultured on aligned scaffolds (Khan et al., 2015; Han et al., 2016; Wanjare et al., 2017). These studies have clearly shown enhanced maturation of hiPSC-CMs cultured on aligned 3D scaffolds based on increased expression of cardiac genes, re-organization of sarcomeres, and an adult cardiomyocyte-like rod-shaped morphology of the cells. Additionally, it has been observed that scaffolds with fibrous, aligned structures mimic the structure of heart tissue, thereby providing a 3D microenvironment for anisotropic arrangement of hiPSC-CMs similar to cardiomyocytes in the heart (Kitsara et al., 2017). Of relevance is another observation made in a previous study by our group (Kumar et al., 2019), wherein hiPSC-CMs cultured in 2D showed similar morphological maturation, but only after 4 weeks in culture. This further reiterates that 3D cultures would be more relevant for structural and functional maturation of hiPSC-CMs, when compared to 2D cultures.

Another important aspect of an *in vitro* model system for cardiac tissue, is the development of a functional syncytium of beating cardiomyocytes. It is a well-established fact that the electrical interconnectivity of cardiomyocytes is an essential pre-requisite for developing *in vitro* cardiac tissues for drug testing applications. This is especially important to determine the effect of a drug molecule on the heart function (e.g., heart rate, arrhythmia-inducing potential; Stoppel et al., 2016; Ugolini et al., 2017). Hence, cell-cell interaction between cardiomyocytes is extremely critical. Previous studies have shown the formation of a functional syncytium in 2D cultures mainly due to hypertrophic growth of hiPSC-CMs (Kumar et al., 2019). On the contrary, functional coupling between cardiomyocytes cultured on 3D constructs has been reported only after mechanical or electrical stimulation (Valls-Margarit et al., 2019). Interestingly, in our study, we observed the formation of a functional syncytium of hiPSC-CMs cultured on the coaxial patches, evidenced by the expression of the gap junction protein, Cx-43, and synchronous calcium transients across the patch. These observations are similar to the behavior of hiPSC-CMs cultured in 2D (Kumar et al., 2019), further indicating that the aligned coaxial scaffolds can be used as an *in vitro* culture system.

It has been shown that 3D culture systems making use of hiPSC-CMs are better suited for cardiac drug testing and toxicity studies because they mimic the *in vivo* response (Kussauer et al., 2019). However, the use of 3D cultures has been restrained due to lack of (a) extensive literature, (b) reproducible culture protocols, and (c) precise analysis tools (Zuppinger, 2019). In recent years, several different techniques have been developed and optimized for analyzing the effect of different cardiac drugs on hiPSC-CMs in 3D cultures, such as calcium imaging using fluorescent dyes, PIV, and measurement of field potentials by MEA (Zuppinger, 2019). In our study, these analysis tools were used to monitor the effectiveness of a few commonly used cardiac drugs on hiPSC-CMs cultured in 2D *vis-à-vis* 3D cultures. As expected, an increase in the beat rate along with a decrease in beat period and FPDc was observed in both the 2D as well as the 3D cultures after ISO treatment. Our observations are consistent with previous studies, where a dose-dependent increase in contractility was observed in 2D cultured hiPSC-CMs following treatment with ISO (Mehta et al., 2011; Blazeski et al., 2012; Kitaguchi et al., 2017), as a result of β1 and β2 receptor stimulation. Additionally, although VER has been known to inhibit voltage-dependent calcium channels (Harris et al., 2013), an increase in contractility was observed in both 2D and 3D cultures. However, consistent with our observation. a recent study identified that this discrepancy in the effect of VER *in vitro* and *in vivo*, resulted from the low potassium concentration in cell culture medium (Zeng et al., 2019). On the other hand, E4031, an hERG channel blocker, resulted in prolonged QT interval (FPDc) in addition to decreased spike amplitude in a dose-dependent manner for 3D cultured cells. The incidence of arrhythmicity increased following the addition of E4031 in both the 2D as well as 3D cultures. However, 3D cultures showed a four-times greater response to drugs than 2D cultures. Our observations are in agreement with earlier reports (Harris et al., 2013) using hiPSC-CMs. Overall, these results strongly indicated an elevated sensitivity of hiPSC-CMs in 3D cultures to various drugs used in this study. Similar observations were reported in the case of 3D culture of primary hepatocytes (Bell et al., 2018; Zhou et al., 2019) and cancer cells (Chaicharoenaudomrung et al., 2019). Likewise, a recent study demonstrated that 3D cardiac spheroids mimics an *in vivo* environment for drug testing and cardiotoxicity studies (Polonchuk et al., 2017).

## Conclusion

Overall, our study demonstrated the fabrication and testing of an aligned coaxial PCL-gelatin nanofiber patch with mechanical and biomimetic properties desired for cardiac applications. hiPSC-CMs cultured on these patches showed a rod-shaped morphology and were aligned in parallel with the nanofibers. These cells on the cardiac patch showed synchronous contractions and exhibited quick response to cardiac drugs. Finally, these cardiac patches can be scaled and used as an *in vitro* drug screening platform for cardiotoxicity studies, as well as to developed into future strategies of cardiac patch transplantation for ischemic heart disease.

## Data Availability Statement

The raw data supporting the conclusions of this article will be made available by the authors, without undue reservation.

## Author Contributions

MK, NK, DS, and AP conceived and designed the experiments. MK, NK, DS, AP, JD, DI, AC, HP, and MM performed the experiments and data analysis. NK, DS, AP, JD, and MM analyzed the data. MK, HP, MA, and AC contributed reagents, materials, and analysis tools. DS, NK, AP, JD, HP, DS, and MK wrote and reviewed the manuscript. The input from all the authors was incorporated in finalizing the manuscript draft.

## Conflict of Interest

The authors declare that the research was conducted in the absence of any commercial or financial relationships that could be construed as a potential conflict of interest.
